# Frequency of Hypomagnesemia and Its Association With Glycemic Control and Diabetic Complications in Adults With Type 2 Diabetes Mellitus

**DOI:** 10.7759/cureus.113353

**Published:** 2026-07-25

**Authors:** Fareeha Salman, Hafiz Zunair Iqbal, Tahseer Khan, Syeda Batool Tirmizi, Omar Naeem, Ehtisham Arif Siddique, Muhammad Anas Ghazi, Ammar Noor, Iqra Naeem, Muhammad Ayoob Memon

**Affiliations:** 1 Diabetes and Endocrinology, Russells Hall Hospital, Dudley, GBR; 2 Internal Medicine, Services Institute of Medical Sciences, Lahore, PAK; 3 Internal Medicine, Akhtar Saeed Medical and Dental College, Lahore, PAK; 4 Internal Medicine, Dow University Hospital, Karachi, PAK; 5 Internal Medicine, King Edward Medical University, Lahore, PAK; 6 Internal Medicine, Pakistan Institute of Medical Sciences, Islamabad, PAK; 7 Trauma and Orthopaedics, Fairfield General Hospital, Bury, GBR; 8 General Surgery, Combined Military Hospital, Lahore, PAK; 9 Internal Medicine, Lahore General Hospital, Lahore, PAK; 10 Psychiatry and Behavioral Sciences, Lahore General Hospital, Lahore, PAK; 11 Internal Medicine, Jinnah Sindh Medical University, Karachi, PAK

**Keywords:** cardiovascular disease, complications, diabetes mellitus, diabetic nephropathy (dn), good glycemic control

## Abstract

Background: Hypomagnesemia is common in patients with type 2 diabetes mellitus (T2DM) and may be related to poor glycemic control and diabetic complications. The present study was conducted to determine the frequency of hypomagnesemia and assess its association with glycemic control, microvascular complications, and macrovascular complications in adults with T2DM.

Methods: This cross-sectional study included 255 adults with T2DM. Serum magnesium was measured in all participants, and hypomagnesemia was defined as serum magnesium <1.6 mg/dL. Patients were divided into hypomagnesemia and normomagnesemia groups. Glycemic parameters, renal indices, lipid profile, microvascular complications, and macrovascular complications were compared between the two groups. Appropriate statistical tests were applied according to data distribution, and multivariable logistic regression was performed to assess adjusted associations.

Results: Hypomagnesemia was identified in 99 (38.8%) patients. Poor glycemic control was significantly more frequent in patients with hypomagnesemia than in those with normomagnesemia (98.0% versus 69.9%; odds ratio (OR) = 20.91; 95% confidence interval (CI), 4.95-88.38; p < 0.001). Microvascular complications were more common among patients with hypomagnesemia, including diabetic retinopathy (47.5% versus 26.3%; OR = 2.54; p < 0.001), diabetic kidney disease (42.4% versus 23.7%; OR = 2.37; p = 0.003), diabetic peripheral neuropathy (41.4% versus 25.6%; OR = 2.05; p = 0.012), and any microvascular complication (71.7% versus 44.2%; OR = 3.20; p < 0.001). Any macrovascular complication was more frequent in the hypomagnesemia group on crude analysis (33.3% versus 21.2%; OR = 1.86; p = 0.044), but this association was not significant after adjustment.

Conclusions: Hypomagnesemia was common among adults with T2DM and was strongly associated with poor glycemic control and microvascular complication burden. Its relationship with macrovascular complications was weaker after adjustment.

## Introduction

Type 2 diabetes mellitus (T2DM) is one of the most common long-term metabolic disorders and remains a major cause of vascular morbidity worldwide. Recent global estimates show that diabetes affected hundreds of millions of adults in 2022, and future projections suggest a further marked rise over the coming decades [1,2]. Pakistan carries a particularly high burden of diabetes, with a large number of adults affected and many patients diagnosed after years of unrecognized disease [3].

Hypomagnesemia is reported more frequently in patients with T2DM than in non-diabetic individuals, although its reported frequency varies widely across different studies [4-6]. This variation is likely related to differences in serum magnesium cut-off values, laboratory methods, dietary intake, renal function, drug exposure, glycemic control, and the clinical setting from which patients are recruited [7,8]. Several recent studies have shown that lower serum magnesium is associated with higher glycated hemoglobin (HbA1c), poor glycemic control, albuminuria, proteinuria, diabetic retinopathy, nephropathy, neuropathy, and foot-related complications [6,9,10]. The relationship with macrovascular disease is less uniform, as some studies have reported associations with coronary heart disease, stroke, peripheral arterial disease, or cardiovascular mortality, while others have found weaker associations after adjustment for age, hypertension, diabetes duration, obesity, renal function, and lipid profile [8,11].

The biological basis of this relationship is clinically relevant. Poor glycemic control may increase urinary magnesium loss through osmotic diuresis, while low magnesium may be associated with impaired insulin signaling, reduced glucose transport, oxidative stress, endothelial dysfunction, and low-grade inflammation [11,12]. However, local evidence remains limited, and available studies differ in sample size, study setting, magnesium cutoff, and range of complications assessed. A recent Pakistani study reported hypomagnesemia in 39% of patients with T2DM and found associations with poor glycemic control and diabetic complications, but further local data are needed in which glycemic control, microvascular complications, and macrovascular complications are assessed together [13]. Therefore, the present study was conducted to determine the frequency of hypomagnesemia and assess its association with glycemic control, microvascular complications, and macrovascular complications in adults with T2DM.

## Materials and methods

After obtaining approval from the Ethical Review Committee (IRB No. 144/RC/KEMU), this cross-sectional design study was conducted in the Department of Medicine, Mayo Hospital, Lahore. This study included 255 patients through non-probability sampling after obtaining written informed consent, and data were collected over a period of six months from December 2024 to June 2025. The sample size was calculated based on the frequency of hypomagnesemia among adults with T2DM as 39%, a margin of error of 6%, and a 95% confidence level; the minimum required sample size was 255 participants [13].

Patients of either sex, aged 30-70 years, with an established diagnosis of T2DM for at least five years were included. Patients were excluded if they had type 1 diabetes mellitus, gestational diabetes, or secondary diabetes due to causes such as chronic pancreatitis, Cushing’s syndrome, or prolonged systemic steroid therapy. Other exclusions were current use of medicines known to affect magnesium balance, including long-term loop or thiazide diuretics, chronic proton pump inhibitor therapy, magnesium-containing laxatives, magnesium supplements, or parenteral magnesium during the preceding three months. Patients with chronic diarrhea, active inflammatory bowel disease, documented malabsorption, recent bariatric surgery, short bowel syndrome, diabetic ketoacidosis, hyperosmolar hyperglycemic state, sepsis, shock, critical illness requiring intensive care, pregnancy, and lactation were not included.

Sociodemographic, lifestyle, clinical, laboratory, and medication history was recorded. A focused examination was performed to evaluate baseline characteristics, including body mass index (BMI) categories, which were interpreted using Asian-specific criteria, blood pressure, and heart rate. Participants were instructed to attend after an overnight fast of at least eight hours. Venous blood samples were drawn for fasting plasma glucose, serum magnesium, serum creatinine, lipid profile, and glycated hemoglobin (HbA1c). Serum magnesium was measured by the hospital laboratory using a standard colorimetric method on an automated analyzer and was reported in mg/dL. Hypomagnesemia was defined as serum magnesium <1.6 mg/dL, while normomagnesemia was defined as 1.6-2.4 mg/dL according to the institutional laboratory reference range. Glycemic control was categorized according to the American Diabetes Association standards, where HbA1c < 7.0% was taken as good glycemic control and HbA1c ≥ 7.0% was taken as poor glycemic control [14]. Renal assessment included serum creatinine, estimated glomerular filtration (eGFR), and urinary albumin-to-creatinine ratio. Diabetic kidney disease was defined as chronic kidney involvement in a patient with type 2 diabetes, documented by a urinary albumin-to-creatinine ratio ≥ 30 mg/g, equivalent albuminuria or proteinuria, and/or eGFR < 60 mL/min/1.73 m² for at least three months, in the absence of another more likely primary renal diagnosis. Diabetic retinopathy was defined as a documented ophthalmologist diagnosis based on dilated fundus examination or retinal imaging, including non-proliferative diabetic retinopathy, proliferative diabetic retinopathy, or diabetic macular edema. Diabetic peripheral neuropathy was defined as distal symmetric peripheral nerve dysfunction documented by compatible symptoms and/or examination findings, such as burning pain, numbness, tingling, reduced vibration or pinprick sensation, loss of protective sensation on 10-g monofilament testing, or reduced ankle reflexes.

Coronary heart disease required a compatible clinical diagnosis supported by objective evidence such as previous myocardial infarction, positive stress testing, coronary angiographic stenosis, or previous percutaneous coronary intervention or coronary artery bypass grafting. Stroke was defined as a documented focal neurological deficit with computed tomography evidence of infarction or intracerebral hemorrhage. Peripheral arterial disease was defined by compatible lower-limb symptoms or ischemic changes supported by ankle-brachial index < 0.90, duplex ultrasound, angiographic evidence of significant stenosis or occlusion, previous lower-limb revascularization, or ischemia-related amputation. Heart failure was recorded when a prior clinical diagnosis was supported by echocardiographic or cardiology documentation.

Data were entered and analyzed using IBM SPSS Statistics for Windows, Version 26 (Released 2019; IBM Corp., Armonk, New York). Quantitative variables were assessed for distributional pattern and were summarized as mean with standard deviation or median with interquartile range, as appropriate. Qualitative variables were presented as frequency and percentage. Comparisons between hypomagnesemia and normomagnesemia groups were performed using the independent samples t-test or Mann-Whitney U test for continuous variables and the chi-square test or Fisher’s exact test for categorical variables. Spearman correlation was used to assess associations between serum magnesium and continuous clinical or biochemical variables. Multivariable logistic regression was applied to adjust selected associations for relevant clinical covariates. A p-value <0.05 was considered statistically significant.

## Results

This cross-sectional study enrolled 255 adults with T2DM. Hypomagnesemia was identified in 99 (38.8%) patients, with 95% CI: 33.0-44.9%. Participants with hypomagnesemia were significantly older (54.00 versus 51.00 years; p < 0.001) and had a longer duration of T2DM (9.90 versus 8.50 years; p = 0.002) than those with normomagnesemia. BMI (29.40 ± 3.61 versus 28.37 ± 4.01 kg/m²; p = 0.033) and systolic blood pressure (135.57 ± 13.79 versus 131.87 ± 14.93 mmHg; p = 0.045) were also significantly higher in the hypomagnesemia group. Physical activity level differed significantly between groups (p = 0.027), with a higher proportion of sedentary participants among those with hypomagnesemia (Table 1).

**Table 1:** Distribution of patient’s characteristics by magnesium status. Continuous variables are presented as mean ± SD or median (IQR) according to distribution and were compared using the independent samples t-test or the Mann-Whitney U test, respectively. Categorical variables are presented as number (%) and were compared using the chi-squared (χ²) test, with Fisher's exact test substituted where an expected cell count was below five. Effect size is reported as the mean/median difference (MD) for continuous variables and odds ratio (OR) for binary categorical variables. T2DM: type 2 diabetes mellitus; BMI: body mass index; BP: blood pressure; DPP-4: dipeptidyl peptidase-4; SGLT2: sodium-glucose cotransporter-2; ACE: angiotensin-converting enzyme; ARB: angiotensin receptor blocker; CVD: cardiovascular disease; CI: confidence interval.

Variable	Total (N = 255)	Hypomagnesemia (n = 99)	Normomagnesemia (n = 156)	Test statistic	Effect size (95% CI)	p-value
Age (years) median (IQR)	53.00 (12.00)	54.00 (10.00)	51.00 (11.00)	U = 9993.0	MD = 3.00	<0.001
Gender, n (%)						
Male	128 (50.2%)	44 (44.4)	84 (53.8)	χ² = 1.78	OR = 0.69 (0.41-1.14)	0.182
Female	127 (49.8%)	55 (55.6)	72 (46.2)			
Residence, n (%)						
Urban	168 (65.9%)	62 (62.6)	106 (67.9)	χ² = 0.54	OR = 0.79 (0.47-1.34)	0.460
Rural	87 (34.1%)	37 (37.4)	50 (32.1)			
Socioeconomic status, n (%)						
High	31 (12.2%)	15 (15.2)	16 (10.3)	χ² = 1.43	-	0.489
Low	61 (23.9%)	22 (22.2)	39 (25.0)			
Middle	163 (63.9%)	62 (62.6)	101 (64.7)			
Smoking status, n (%)						
Current	50 (19.6%)	16 (16.2)	34 (21.8)	χ² = 1.24	-	0.538
Former	28 (11.0%)	11 (11.1)	17 (10.9)			
Never	177 (69.4%)	72 (72.7)	105 (67.3)			
Physical activity, n (%)						
Mild	96 (37.6%)	36 (36.4)	60 (38.5)	χ² = 9.16	-	0.027
Moderate	55 (21.6%)	13 (13.1)	42 (26.9)			
Regular exercise	31 (12.2%)	15 (15.2)	16 (10.3)			
Sedentary	73 (28.6%)	35 (35.4)	38 (24.4)			
Family history of diabetes n (%)	137 (53.7%)	54 (54.5)	83 (53.2)	χ² = 0.01	OR = 1.06 (0.64-1.75)	0.936
Family history of CVD n (%)	88 (34.5%)	41 (41.4)	47 (30.1)	χ² = 2.93	OR = 1.64 (0.97-2.77)	0.087
Duration of T2DM (years) median (IQR)	8.90 (6.15)	9.90 (6.40)	8.50 (5.65)	U = 9513.5	MD = 1.40	0.002
Current treatment, n (%)						
Insulin	26 (10.2%)	9 (9.1)	17 (10.9)	χ² = 4.51	-	0.212
Lifestyle only	15 (5.9%)	6 (6.1)	9 (5.8)			
Oral drugs	166 (65.1%)	59 (59.6)	107 (68.6)			
Oral drugs + insulin	48 (18.8%)	25 (25.3)	23 (14.7)			
Metformin use n (%)	189 (74.1%)	73 (73.7)	116 (74.4)	χ² = 0.00	OR = 0.97 (0.55-1.72)	1.000
Sulfonylurea use n (%)	86 (33.7%)	26 (26.3)	60 (38.5)	χ² = 3.51	OR = 0.57 (0.33-0.99)	0.061
DPP-4 inhibitor use n (%)	52 (20.4%)	17 (17.2)	35 (22.4)	χ² = 0.74	OR = 0.72 (0.38-1.36)	0.391
SGLT2 inhibitor use n (%)	28 (11.0%)	14 (14.1)	14 (9.0)	χ² = 1.17	OR = 1.67 (0.76-3.67)	0.280
Insulin use n (%)	74 (29.0%)	34 (34.3)	40 (25.6)	χ² = 1.82	OR = 1.52 (0.88-2.63)	0.177
Treatment adherence, n (%)						
Good	72 (28.2%)	25 (25.3)	47 (30.1)	χ² = 5.78	-	0.056
Partial	146 (57.3%)	65 (65.7)	81 (51.9)			
Poor	37 (14.5%)	9 (9.1)	28 (17.9)			
BMI (kg/m²) mean ± SD	28.77±3.89	29.40 ± 3.61	28.37 ± 4.01	t = 2.14	MD = 1.04 (0.09-1.99)	0.033
Systolic BP (mmHg) mean ± SD	133.31±14.58	135.57 ± 13.79	131.87 ± 14.93	t = 2.02	MD = 3.69 (0.11-7.28)	0.045
Diastolic BP (mmHg) mean ± SD	78.20±7.50	77.90 ± 8.27	78.40 ± 7.00	t = -0.50	MD = -0.50 (-2.46-1.47)	0.619
Hypertension n (%)	147 (57.6%)	64 (64.6)	83 (53.2)	χ² = 2.80	OR = 1.61 (0.96-2.70)	0.095
Dyslipidemia n (%)	134 (52.5%)	57 (57.6)	77 (49.4)	χ² = 1.33	OR = 1.39 (0.84-2.31)	0.249
Statin use n (%)	160 (62.7%)	66 (66.7)	94 (60.3)	χ² = 0.81	OR = 1.32 (0.78-2.23)	0.369
ACE inhibitor/ARB use n (%)	155 (60.8%)	68 (68.7)	87 (55.8)	χ² = 3.72	OR = 1.74 (1.02-2.95)	0.054

Fasting plasma glucose (173.00 (38.50) versus 144.00 (39.25) mg/dL; p < 0.001) and HbA1c (9.00 (1.70) versus 7.50% (1.30); p < 0.001) were significantly higher in participants with hypomagnesemia, and poor glycemic control was correspondingly more frequent in this group (98.0% versus 69.9%; OR = 20.91, 95% CI: 4.95-88.38; p < 0.001). Renal function was also poorer in the hypomagnesemia group, reflected in higher serum creatinine (p < 0.001), lower eGFR (70.83 ± 19.62 versus 82.46 ± 19.13 mL/min/1.73m²; p < 0.001), and a higher urinary albumin-to-creatinine ratio (p < 0.001). Among lipid parameters, only HDL cholesterol differed significantly, being lower in the hypomagnesemia group (41.00 (12.50) versus 43.00 (12.25) mg/dL; p = 0.029); total cholesterol, LDL cholesterol, and triglycerides did not differ significantly (all p > 0.05) (Table 2).

**Table 2:** Glycemic, renal, and lipid biochemical parameters by magnesium status. Continuous variables are presented as mean ± SD or median (IQR) according to distribution and were compared using the independent samples t-test or the Mann-Whitney U test, respectively. Categorical variables are presented as number (%) and were compared using the chi-squared (χ²) test. Effect size is reported as the mean/MD for continuous variables and OR for binary categorical variables. HbA1c: glycated hemoglobin; eGFR: estimated glomerular filtration rate; UACR: urinary albumin-to-creatinine ratio; LDL: low-density lipoprotein; HDL: high-density lipoprotein; CI: confidence interval.

Variable	Total (N = 255)	Hypomagnesemia (n = 99)	Normomagnesemia (n = 156)	Test statistic	Effect size (95% CI)	p-value
Fasting plasma glucose (mg/dL) median (IQR)	157.00 (45.00)	173.00 (38.50)	144.00 (39.25)	U = 11686.5	MD = 29.00	<0.001
HbA1c median (IQR)	7.90 (1.65)	9.00 (1.70)	7.50 (1.30)	U = 12873.5	MD = 1.50	<0.001
Poor glycemic control n (%)	206 (80.8%)	97 (98.0)	109 (69.9)	χ² = 29.04	OR = 20.91 (4.95-88.38)	<0.001
Good glycemic control n (%)	49 (19.2%)	2 (2.0)	47 (30.1)			
Serum creatinine (mg/dL) median (IQR)	0.80 (0.25)	0.85 (0.29)	0.78 (0.18)	U = 9829.0	MD = 0.07	<0.001
eGFR (mL/min/1.73 m²) mean ± SD	78.80 (23.20)	70.83 ± 19.62	82.46 ± 19.13	t = -4.65	MD = -11.62 (-16.51, -6.73)	<0.001
UACR (mg/g) median (IQR)	19.00 (41.35)	28.60 (90.70)	14.70 (21.52)	U = 10475.5	MD = 13.90	<0.001
Total cholesterol (mg/dL) mean ± SD	187.00 (46.00)	189.88 ± 37.46	187.80 ± 35.83	t = 0.44	MD = 2.08 (-7.20, 11.35)	0.661
LDL cholesterol (mg/dL) median (IQR)	101.00 (53.50)	100.00 (62.00)	102.00 (44.25)	U = 7744.0	MD = -2.00	0.970
HDL cholesterol (mg/dL) median (IQR)	42.36 ± 8.76	41.00 (12.50)	43.00 (12.25)	U = 6472.0	MD=-2.00	0.029
Triglycerides (mg/dL) median (IQR)	170.00 (98.50)	170.00 (87.00)	170.00 (109.75)	U = 6940.5	MD=0.00	0.174

Vascular complications were more frequent in the hypomagnesemia group. Individual macrovascular outcomes, including coronary heart disease, stroke, peripheral arterial disease, and heart failure, showed no significant group-wise difference, although any macrovascular complication was higher with hypomagnesemia (33.3% versus 21.2%; OR = 1.86, 95% CI: 1.06-3.29; p = 0.044). Microvascular disease showed a clearer pattern, with significantly higher retinopathy, diabetic kidney disease, and peripheral neuropathy in hypomagnesemic patients. Any microvascular complication was also more frequent in this group (71.7% versus 44.2%; OR = 3.20, 95% CI: 1.86-5.48; p < 0.001) (Table 3).

**Table 3:** Macrovascular and microvascular complications by magnesium status. Categorical variables are presented as number (%) and were compared using the chi-squared (χ²) test. OR: odds ratio; CI: confidence interval.

Variable	Total (N = 255) n (%)	Hypomagnesemia (n = 99) n (%)	Normomagnesemia (n = 156) n (%)	Test statistic	OR (95% CI)	p-value
Coronary heart disease	51 (20.0)	26 (26.3)	25 (16.0)	3.35	1.87 (1.00-3.47)	0.067
Stroke	18 (7.1)	9 (9.1)	9 (5.8)	0.58	1.63 (0.63-4.27)	0.448
Peripheral arterial disease	27 (10.6)	14 (14.1)	13 (8.3)	1.59	1.81 (0.81-4.04)	0.208
Heart failure	21 (8.2)	10 (10.1)	11 (7.1)	0.40	1.48 (0.60-3.63)	0.529
Any macrovascular complication	66 (25.9)	33 (33.3)	33 (21.2)	4.07	1.86 (1.06-3.29)	0.044
Diabetic retinopathy	88 (34.5)	47 (47.5)	41 (26.3)	11.12	2.54 (1.49-4.31)	<0.001
Diabetic kidney disease	79 (31.0)	42 (42.4)	37 (23.7)	9.06	2.37 (1.38-4.08)	0.003
Diabetic peripheral neuropathy	81 (31.8)	41 (41.4)	40 (25.6)	6.24	2.05 (1.20-3.51)	0.012
Any microvascular complication	140 (54.9)	71 (71.7)	69 (44.2)	17.39	3.20 (1.86-5.48)	<0.001

Four multivariable logistic regression models were constructed to evaluate the independent associations among hypomagnesemia, glycemic control, and vascular complication status, with mutual adjustment for age, sex, duration of diabetes, BMI, and hypertension (Table 4). Hypomagnesemia and poor glycemic control showed a strong, mutually independent association in both directions (Model 1: AOR = 15.35, 95% CI: 3.52-66.86, p < 0.001 for poor glycemic control predicting hypomagnesemia; Model 2: AOR = 15.81, 95% CI: 3.68-67.97, p < 0.001 for the reverse); age was an independent predictor in both models (p = 0.007 and p = 0.042, respectively). Any microvascular complication independently predicted hypomagnesemia (Model 1: AOR = 2.72, 95% CI: 1.47-5.03, p = 0.001), but the reverse association was attenuated and non-significant after adjustment (Model 3: AOR = 1.69, 95% CI: 0.79-3.61, p = 0.177), where male sex (AOR = 2.78, p = 0.001) and eGFR (AOR = 0.96, p < 0.001) emerged as significant independent predictors instead. In Model 4, no variable, including hypomagnesemia (AOR = 1.45, 95% CI: 0.70-3.02, p = 0.321), was independently associated with any macrovascular complication, and the overall model did not reach significance (p = 0.159).

**Table 4:** Multivariable logistic regression models. The Wald z statistic tested the significance of each regression coefficient. Model 1 (n = 255): χ² = 73.43; p < 0.001; R² = 0.216. Model 2 (n = 255): χ² = 47.65; p < 0.001; R² = 0.191. Model 3 (n = 255): χ² = 69.83; p < 0.001; R² = 0.199. Model 4 (n = 255): χ² = 10.56; p = 0.159 (not statistically significant); R² = 0.036. AOR: adjusted odds ratio; CI: confidence interval.

Model/predictor	AOR	95% CI	Wald z	p-value
Outcome = Hypomagnesemia				
Poor glycemic control	15.35	3.52-66.86	3.64	<0.001
Any microvascular complication	2.72	1.47-5.03	3.18	0.001
Any macrovascular complication	1.65	0.85-3.21	1.47	0.142
Age, years	1.06	1.01-1.10	2.70	0.007
Male	0.77	0.42-1.41	-0.85	0.397
Duration of diabetes, years	1.06	0.99-1.13	1.66	0.098
BMI, kg/m²	1.04	0.96-1.12	0.90	0.366
Hypertension	1.28	0.70-2.35	0.80	0.422
Outcome = Poor Glycemic Control				
Hypomagnesemia	15.81	3.68-67.97	3.71	<0.001
Age, years	1.05	1.00-1.10	2.04	0.042
Male	0.76	0.37-1.54	-0.77	0.441
Duration of diabetes, years	1.06	0.97-1.15	1.32	0.185
BMI, kg/m²	1.05	0.96-1.15	1.11	0.266
Hypertension	0.85	0.42-1.71	-0.46	0.649
Outcome = Any Microvascular Complication				
Hypomagnesemia	1.69	0.79-3.61	1.35	0.177
Age, years	1.03	0.99-1.07	1.68	0.092
Male	2.78	1.50-5.12	3.27	0.001
Duration of diabetes, years	1.07	1.00-1.15	1.89	0.058
HbA1c, percent	1.12	0.84-1.49	0.74	0.457
Hypertension	1.76	0.97-3.17	1.87	0.062
eGFR, mL/min/1.73m²	0.96	0.94-0.97	-4.97	<0.001
Outcome = Any Macrovascular Complication				
Hypomagnesemia	1.45	0.70-3.02	0.99	0.321
Age, years	1.03	0.99-1.07	1.40	0.160
Male	1.34	0.75-2.42	0.99	0.323
Duration of diabetes, years	1.07	1.00-1.14	1.87	0.061
HbA1c, percent	1.04	0.79-1.38	0.30	0.761
BMI, kg/m²	0.99	0.92-1.07	-0.14	0.886
Hypertension	0.91	0.51-1.66	-0.29	0.769

Serum magnesium showed significant negative correlations with HbA1c (r = -0.442, p < 0.001), fasting plasma glucose (r = -0.335, p < 0.001), serum creatinine (r = -0.279, p < 0.001), urinary albumin-to-creatinine ratio (r = -0.269, p < 0.001), duration of diabetes (r = -0.235, p < 0.001), and age (r = -0.205, p = 0.001). It correlated positively with eGFR (r = 0.263, p < 0.001). Weak positive correlations were observed with triglycerides and HDL cholesterol, while BMI, blood pressure, total cholesterol, and LDL cholesterol were not significantly correlated (Table 5).

**Table 5:** Correlation of serum magnesium level with continuous clinical and biochemical variables. r: Spearman's rank correlation coefficient; CI: confidence interval; eGFR: estimated glomerular filtration rate; UACR: urinary albumin-to-creatinine ratio; LDL: low-density lipoprotein; HDL: high-density lipoprotein; HbA1c: glycated hemoglobin.

Variable	r	95% CI	p-value
Age (years)	-0.20	-0.32-0.08	0.001
Duration of type 2 diabetes mellitus (years)	-0.23	-0.35 to -0.12	<0.001
Body mass index (kg/m²)	-0.10	-0.22-0.03	0.119
Systolic blood pressure (mmHg)	-0.06	-0.18-0.07	0.375
Diastolic blood pressure (mmHg)	-0.01	-0.13-0.12	0.924
Fasting plasma glucose (mg/dL)	-0.34	-0.44 to -0.22	<0.001
HbA1c (%)	-0.44	-0.54 to -0.34	<0.001
Serum creatinine (mg/dL)	-0.28	-0.39 to -0.16	<0.001
eGFR (mL/min/1.73 m²)	0.26	0.14-0.37	<0.001
Urinary albumin-to-creatinine ratio (mg/g)	-0.27	-0.38 to -0.15	<0.001
Total cholesterol (mg/dL)	-0.01	-0.14-0.11	0.814
LDL cholesterol (mg/dL)	0.00	-0.12-0.13	0.948
HDL cholesterol (mg/dL)	0.13	0.01-0.25	0.040
Triglycerides (mg/dL)	0.14	0.02-0.26	0.022

## Discussion

The present study identified hypomagnesemia in 38.8% of patients, identical to the 39% reported in a Pakistani hospital-based sample using the same <1.6 mg/dL cut-off, supporting consistency with local data [13]. Comparable South Asian studies reported frequencies of 34% and 30%, suggesting that the present estimate fits within the expected range for hospital-based diabetic populations [15,16]. Lower estimates from Palestine and Turkey, 11.0% and 9.7%, respectively, may reflect primary-care sampling, different case mix, better metabolic control, or laboratory classification [4,17]. The observed frequency is supported by international heterogeneity in magnesium cutoffs, assay methods, renal status, and population selection [11,18,19].

Participants with hypomagnesemia were older and had longer diabetes durations than those with normomagnesemia, and serum magnesium was reported to have negative correlations with both age and duration of diabetes. This pattern is consistent with studies in which older age and longer duration of diabetes were linked with lower magnesium status, suggesting that magnesium deficiency is more often observed in patients with longer metabolic exposure rather than in newly diagnosed or better-controlled diabetic populations [11,20]. A possible biological explanation is that prolonged hyperglycemia may be accompanied by osmotic diuresis, renal magnesium loss, and impaired insulin-sensitive tubular magnesium handling through TRPM6 channels, while increasing disease duration may add cumulative renal and metabolic stress [11,21].

BMI and systolic blood pressure were also higher in the hypomagnesemia group, although BMI was not significantly correlated with serum magnesium and hypertension did not differ significantly between groups. This suggests that adiposity and blood pressure may contribute to an adverse metabolic profile but were not the strongest direct correlates of magnesium level in this sample. Similar inconsistency has been described in previous studies, where obesity was sometimes associated with lower magnesium status, whereas hypertension did not show a stable association across all diabetic samples [13,18]. The higher BMI in hypomagnesemia patients may be related to insulin resistance, inflammatory burden, and poorer dietary quality, but the non-significant correlation indicates that glycemic status and renal indices may have been more influential in the present data [18,22].

The strongest comparative finding was poorer glycemic control in the hypomagnesemia group, with higher fasting plasma glucose, higher HbA1c, and markedly greater frequency of poor glycemic control. This agrees with previous studies showing lower magnesium among patients with higher HbA1c or poorer glycemic categories [4,5,23,24]. The negative correlation between magnesium and HbA1c supports a bidirectional relationship, where poor glycemic control may increase renal magnesium wasting, while low magnesium may be associated with impaired insulin secretion and insulin receptor signaling [9,12,21].

Microvascular complications were more frequent among participants with hypomagnesemia in the present study, with significant differences observed for diabetic retinopathy, diabetic kidney disease, diabetic peripheral neuropathy, and the composite microvascular outcome. The finding for retinopathy is consistent with local and regional evidence, as retinopathy was reported more often among hypomagnesemic patients in Pakistani and Indian diabetic samples, with frequencies close to the present estimate in some studies [13,15,16]. The magnitude of association in the present study was also comparable with the adjusted association reported in Palestinian patients, where hypomagnesemia was linked with retinopathy after adjustment for relevant variables [4]. Lower serum magnesium in patients with retinopathy and still lower levels in sight-threatening retinopathy suggest that magnesium status may be related not only to the presence of retinal disease but also to its severity, although the present study did not grade retinopathy severity [5,25].

The renal findings were also consistent with previous literature, as patients with hypomagnesemia had higher serum creatinine, lower eGFR, higher UACR, more proteinuria, and greater frequency of diabetic kidney disease. Similar associations of low magnesium with nephropathy, albuminuria, and urinary protein have been described in Pakistani, Turkish, and other diabetic populations [13,17,26]. The renal association appears biologically reasonable because hyperglycemia, tubular dysfunction, and altered magnesium handling may occur together in diabetic kidney involvement, while reduced renal reserve may also influence serum magnesium interpretation [11]. This relationship remains complex because advanced renal impairment may be accompanied by magnesium retention rather than deficiency, which may explain differences between studies that included patients with more severe kidney disease and the present study, where severe renal impairment was excluded [12,17].

Peripheral neuropathy was also significantly more frequent in the hypomagnesemia group, which is supported by studies reporting more neuropathy among patients with low magnesium [5,17]. Evidence based on nerve conduction testing has also shown lower magnesium in patients with impaired peripheral nerve function, supporting the clinical association observed in the present study. Differences between studies may reflect variation in neuropathy assessment, as clinical symptoms, monofilament testing, and nerve conduction studies do not identify identical patient groups [8,15,17]. Overall, the higher composite microvascular burden in the hypomagnesemia group is consistent with the shared pathways of poor glycemic control, renal dysfunction, oxidative stress, endothelial injury, and inflammatory activity described in previous literature [11,12].

Macrovascular findings in the present study were less consistent than the glycemic and microvascular findings. Coronary heart disease, stroke, peripheral arterial disease, and heart failure were numerically more frequent in the hypomagnesemia group, but none reached statistical significance individually. The composite macrovascular outcome was significant in crude analysis, yet hypomagnesemia was not independently associated with macrovascular complications after adjustment, suggesting that the crude difference was partly explained by age, diabetes duration, BMI, hypertension, and vascular risk factors. This pattern agrees with studies in which unadjusted associations between serum magnesium and coronary disease, stroke, or peripheral arterial disease weakened after adjustment for cardiovascular confounders [11,12,22,27].

The present finding also fits the mixed primary-study evidence. Some studies reported higher coronary heart disease, cerebrovascular accident, or peripheral arterial disease among patients with low magnesium, supporting the direction of the crude association seen here [5,21,24]. Other studies found either similar coronary disease rates between magnesium groups or non-significant macrovascular differences, which resemble the adjusted findings of the present analysis [4,13]. The significant association only among hypertensive participants is clinically plausible, because magnesium status has been linked with vascular tone, aldosterone activity, oxidative stress, nitric oxide availability, and inflammatory pathways overlapping with hypertensive vascular disease [11].

After adjustment and stratified analysis, hypomagnesemia appeared to be mainly associated with poor glycemic control and microvascular complications, whereas its association with macrovascular disease was less consistent. This finding is clinically plausible, as chronic hyperglycemia can increase urinary magnesium loss, while magnesium deficiency may impair insulin secretion and insulin action, further worsening glycemic control [9,11,12]. The weaker association with vascular complications after adjustment suggests that the observed relationship was partly shared with other factors, including diabetes duration, renal function, hypertension, and long-term metabolic exposure [13,17,28]. Differences in magnesium cutoffs, renal eligibility criteria, medication exclusions, and definitions of diabetic complications may also explain variation across studies [25,29,30]. Therefore, these findings should be interpreted as associations rather than evidence of causation.

The study has important strengths, including an adequate sample size, complete assessment of all enrolled patients, and evaluation of multiple variables with appropriate statistical adjustment. However, its cross-sectional design limits assessment of temporal direction. This single-center study used consecutive sampling, so results may not reflect the wider diabetic population. Magnesium was measured once as total serum levels; other forms and related factors were not assessed. The sample size was set to estimate hypomagnesemia, not for detailed outcome analysis, so some results need caution. Kidney findings had overlap in measures used. Insulin resistance was not directly assessed, and multiple comparisons may have increased the chance of random associations.

## Conclusions

Hypomagnesemia was common among adults with T2DM in this study. Patients with low serum magnesium had poorer glycemic control and a higher burden of microvascular complications. Macrovascular complications were more frequent in patients with hypomagnesemia on initial comparison, but this relationship was weaker after adjustment for other clinical factors. These findings suggest that serum magnesium is closely linked with metabolic control and microvascular disease profile in T2DM.
